# Cosmetic Applications of Bee Venom

**DOI:** 10.3390/toxins13110810

**Published:** 2021-11-18

**Authors:** Aida A. Abd El-Wahed, Shaden A. M. Khalifa, Mohamed H. Elashal, Syed G. Musharraf, Aamer Saeed, Alfi Khatib, Haroon Elrasheid Tahir, Xiaobo Zou, Yahya Al Naggar, Arshad Mehmood, Kai Wang, Hesham R. El-Seedi

**Affiliations:** 1 Department of Bee Research, Plant Protection Research Institute, Agricultural Research Centre, Giza 12627, Egypt; aidaabd.elwahed@arc.sci.eg; 2 Department of Molecular Biosciences, The Wenner-Gren Institute, Stockholm University, S-106 91 Stockholm, Sweden; 3 Department of Chemistry, Faculty of Science, Menoufia University, Shebin El-Kom 32512, Egypt; m_h_elashal@yahoo.com; 4 H.E.J. Research Institute of Chemistry, International Center for Chemical and Biological Sciences, University of Karachi, Karachi 75270, Pakistan; musharraf@iccs.edu; 5 Department of Chemistry, Quaid-i-Azam University, Islamabad 45320, Pakistan; asaeed@qau.edu.pk; 6 Pharmacognosy Research Group, Department of Pharmaceutical Chemistry, Kulliyyah of Pharmacy, International Islamic University Malaysia, Kuantan 25200, Pahang Darul Makmur, Malaysia; alfikhatib@iium.edu.my; 7 Faculty of Pharmacy, Airlangga University, Surabaya 60155, Indonesia; 8 School of Food and Biological Engineering, Jiangsu University, Zhenjiang 212013, China; haroona28@yahoo.com (H.E.T.); zou_xiaobo@ujs.edu.cn (X.Z.); 9 General Zoology, Institute for Biology, Martin Luther University Halle-Wittenberg, Hoher Weg 8, 06120 Halle, Germany; yehia.elnagar@science.tanta.edu.eg; 10 Zoology Department, Faculty of Science, Tanta University, Tanta 31527, Egypt; 11 Beijing Engineering and Technology Research Center of Food Additives, Beijing Technology and Business University, Beijing 100048, China; arshadfst@yahoo.com; 12 Institute of Apicultural Research, Chinese Academy of Agricultural Sciences, Beijing 100093, China; 13 Pharmacognosy Group, Department of Pharmaceutical Biosciences, Uppsala University, Biomedical Centre, Box 591, SE-751 24 Uppsala, Sweden; 14 International Research Center for Food Nutrition and Safety, Jiangsu University, Zhenjiang 212013, China

**Keywords:** bee venom, cosmetics applications, skin diseases

## Abstract

Bee venom (BV) is a typical toxin secreted by stingers of honeybee workers. BV and BV therapy have long been attractive to different cultures, with extensive studies during recent decades. Nowadays, BV is applied to combat several skin diseases, such as atopic dermatitis, acne vulgaris, alopecia, vitiligo, and psoriasis. BV is used extensively in topical preparations as cosmetics and used as dressing for wound healing, as well as in facemasks. Nevertheless, the safety of BV as a therapeutic choice has always been a concern due to the immune system reaction in some people due to BV use. The documented unfavorable impact is explained by the fact that the skin reactions to BV might expand to excessive immunological responses, including anaphylaxis, that typically resolve over numerous days. This review aims to address bee venom therapeutic uses in skin cosmetics.

## 1. Introduction

Bee venom (BV) includes a complex mixture of peptides, enzymes, lipids, and bioactive amines, and the accumulating body of data indicates its wide variety of pharmaceutical properties [1]. Accordingly, the BV remedy has been developed to deal with various sicknesses, including inflammation, cancers, microbial diseases, and neurodegenerative diseases [1,2,3,4]. Recently, BV has been used as a topical treatment for atopic dermatitis and acne because of its anti-inflammatory, anti-ageing [5,6], and anti-bacterial properties [7]. BV can be useful as a topical agent for encouraging skin regeneration and thus treatment of certain epidermal conditions [6,7]. BV’s topical administration can be well tolerated in human skin as it has shown no risk for dermal irritation in animal studies [8]. It is widely marketed in Europe under the names of Forapin in Germany, Virapin in Slovakia, Apiven in France, Melivenon in Bulgaria, and Apifor in Russia [9].

Allergic reactions remain the principal challenges facing the usual approval and application of BV. The venom hypersensitivity can be fatal if developed to intense systemic allergy (SAR) [10]. In this context, venom immunotherapy (VIT) is the exceptional treatment of bee sting-induced systemic hypersensitive reactions [11,12,13].

In this review, we discuss the applications of BV as cosmetic products for skin diseases.

## 2. Cosmetic Applications of Bee Venom

**Figure 1:**
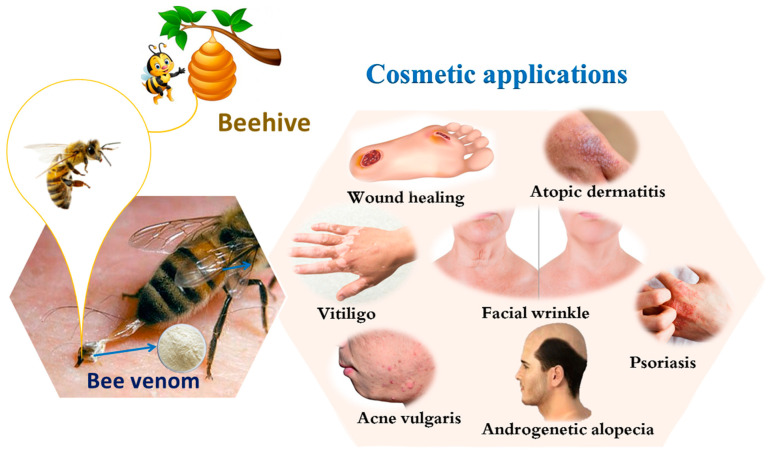
Cosmetic applications of bee venom for skin diseases.

The biological properties of BV have recently encouraged researchers to use it in cosmetic applications, as explained below in detail and as summarized in Figure 1.

### 2.1. Atopic Dermatitis (AD)

Atopic dermatitis (AD) is a devastating skin disease affecting up to 230 million worldwide, with the highest burden in childhood [14]. AD is common in Western countries, and treatment options are limited, especially since the main cause is unknown. Several causes, such as inflammatory and immunological insults, can cause AD [15]. Many commercial drugs such as topical corticosteroids, dupilumab, Janus kinase inhibitors, calcineurin inhibitors, and cyclosporine A have been developed for to calm the AD patient’s skin. However, they are often associated with severe local and systemic side effects, including neurotoxicity, infections, skin cancers, and the disruption of the epidermal permeability barrier [16,17,18]. Therefore, several studies on herbs and venoms for curing AD have been conducted [19].

AD was induced in Balb/c mice by the topical application of 5% phthalic anhydride (PA). After 24 h, BV was applied to the mice’s back and ear skin at doses of 0.3 mg/kg 3 times/week for 4 weeks. BV decreases the activation of nuclear factor κB (NF-κB) and MAP kinase and leads to a reduced expression of tumor necrosis factor-α (TNF-α), interleukin-1β (IL-1β) inducible nitric oxide (iNO), and cyclooxygenase-2 (COX-2), thus diminishing the skin damage [20]. BV treatment was found to significantly alleviate AD clinical features such as the thickness of the ear skin, inflammation, and lymph node enlargement. Upregulation of p50, a decrease of the quantity of mast cells, and a less epidermal thickness were observed within the BV-treated groups compared to the PA-treated group [21,22]. Similarly, bilateral subcutaneous acupuncture injections of BV (0.3 mg/kg/day for 5 days) behind the knee in a mouse model manifested with atopy-like dermatitis significantly inhibited the expression of both T helper cell type 1 (Th1) and Th2 cytokines. Atopic dermatitis was induced by patching ovalbumin (OVA), and the OVA-induced skin thickening and inflammatory infiltration were decreased in the BV-treated group, supporting the BV role as an anti-inflammatory agent [23]. The intraperitoneal administration of some BV compounds (48/80) (0.01 and 0.1 mg/kg) inhibited mast cell degranulation and the formation of pro-inflammatory cytokines in skin tissues [24]. Additionally, apamin (isolated from bee venom) has a therapeutic anti-inflammatory effect on AD, especially at a 0.1 μg/mL concentration, by suppressing inflammatory cytokines and chemokines. For instance, apamin administration blocked the activation of signal transducer and activator of transcription (STAT-1, STAT-3) and NF-κB, the known transcription factors in keratinocytes [25].

In a clinical study, 136 patients with atopic dermatitis were randomized to receive either an emollient containing BV and silk protein or a vehicle for 4 weeks. Patients with emollients containing BV showed significant lower severity index score and visual analogue scale value compared to patients in the control group. Irritation, pruritus, erythema, urticaria, and disease worsening were frequently reported as unfavorable “side effects,” according to the study. These were mild reactions that did not interfere with daily activities and did not require treatment [26].

BV and/or melittin (100, 200, and 500 μg mixed with normal saline) was applied 5 times/week for 4 weeks to the dorsal skin of the mice model. BV and melittin decreased AD-like skin lesions induced by 2,4-dinitrochloroben-zene. BV and melittin decreased the expression of chemokines, such as CCL17 and CCL22, and pro-inflammatory cytokines, including IL-1β, IL-6, and IFN-γ, through the blockage of the NF-κB and STAT signaling pathways in an in vitro study using TNF-α/IFN-γ-stimulated human keratinocytes [27].

Moreover, bvPLA2 (16 and 80 ng/ear), another active component of BV, was applied with a brush 4 times/week for 3 weeks to a mice model where dexamethasone (50 µg/ear) was used as a positive control. The elevated levels of serum immunoglobulin E (IgE), as well as Th1 and Th2 cytokines, were considerably decreased after the administration of bvPLA2. The intake of bvPLA2 reduced the histological alterations and specifically the mast cell infiltration. BV and its components could be promising candidates for treating AD [28].

### 2.2. Acne Vulgaris

Acne vulgaris is a dermatological lesion that mainly affects teenagers, affecting women at higher rates than men [29]. Acne is usually distributed on the face, arms, chest, or back [30]. Acne manifests as increased sebum production, ductal cornification, pilosebaceous canal bacterial colonization, and inflammation [31]. Antibiotics are normally given for acne vulgaris primarily to kill the bacteria and suppress inflammation. However, advancing antibiotic resistance is of global concern, and the restricted use of antibiotics tends to be a more environmentally friendly option [32,33]. Naturally occurring products with antimicrobial properties have commonly been used for primary acne care. *Propionibacterium acnes* is a Gram-positive bacterium that is responsible for the inflammatory reactions in acne vulgaris by stimulating inflammatory cells. In vitro studies suggested thatHaCaT, and monocytes (THP-1) could secrete pro-inflammatory cytokines, such as IL-1β, IL-8, tumor necrosis factors (TNF-α, IFNγ), and toll-like receptor 2 (TLR2) after stimulated with *P. acnes* [34,35,36,37]. BV blocked the expression of TLR2 and inhibited the development of *P. acnes*-induced IFN-π, IL-1β, IL-8, and TNF-α in HaCaT and in THP-1 cells [38]. The *P. acnes*-injected mice treated with BV/Vaseline cocktail (1 μg/0.05 g) displayed a significant decrease in skin thickness, swelling, erythema, and inflammatory reactions compared to the untreated group. In addition to inhibition of TLR2 and CD14 expressions, the expression levels of TNF-α and IL-1β were significantly reduced in the BV-treated mice. Upon treatment with the venom, the binding activity of the NF-κB and activator protein (AP)-1 was significantly suppressed [39]. BV serum (purified BV diluted in cold sterile water) was administered twice daily for 3 to 6 weeks and was found to stimulate the healing progress from 8.6% to 52.3% in 30 volunteers with mild to moderate acne vulgaris, bearing in mind that they had no previous experience with the skin care regimen [40]. It was noted that cosmetics containing BV offered a degree of effectiveness greater than those without BV [41]. For instance, applying BV-containing care products twice daily led to better management and preferable outcomes in terms of reducing of both inflammatory and non-inflammatory lesions and decreasing skin microorganisms [38]. Because the irritating potential of BV is low, long-term usage of cosmetics containing BV therefore could be considered safe.

### 2.3. Androgenetic Alopecia (AGA)

Androgenetic alopecia (AGA) is one of the most frequent chronic skin problems. It is characterized by progressive hair loss, particularly scalp hair, with distinctive loss trends in women and men; however, the central scalp is most severely affected in both genders [42]. AGA is an age-related illness that affects more than 80% and 42% of Caucasian men and women, respectively, at the age of 70, but commonly begins during puberty. Several causes are thought to cause alopecia, such as reduced growth and regeneration of fibroblast follicle cells, increased 5α-dihydrotestosterone, and chemotherapy. Two manufactured medicines, topical minoxidil and oral finasteride, have been endorsed by the Food and Drug Administration (FDA) in addition to various non-prescription drugs claimed to be effective in AGA hair restoration to date [43,44]. However, these two medications have poor cure rates (<50%) and several side effects, including skin irritation, allergic contact dermatitis, and increased body hair growth or darkening [45]. Therefore, alternative medicines with improved efficacy and safety are needed. In C57BL/6 female mice, local administration of 0.01% BV improved hair growth. BV enhances hair follicle development by reducing expression of 5α-reductase; stimulates expression of growth factors, such as VEGF, insulin-like growth factor 1 (IGH-1), fibroblast growth factor 7 (FGF7), and fibroblast growth factor 2 (FGF2); and impedes the catagen process. BV also improves human dermal papilla cell proliferation (hDPC) relative to a positive control (2% minoxidil). BV did not cause erythema, oedema, inflammation, or cytotoxicity with the concentration used (Table 1) [46].

### 2.4. Wound Healing

Wound healing is a dynamic and complex tissue repair process involving various molecular and cellular events. The healing starts with an inflammatory response and ends with re-epithelialization and, eventually, a permanent scar [47]. BV, with its anti-inflammatory, anti-microbial, analgesic, and antioxidant properties, has a great potential for wound healing. The usage of 6% BV-chitosan films as topical formulations for rapid complete sterile wound healing in diabetic rats was satisfying and compatible with the skin. This combination has a better anti-inflammatory effect than chitosan film solely (Table 1) [48]. BV treatment dramatically enhanced the closure of wounds in diabetic mice by increasing the collagen and *β*-defensin-2 (BD-2) expression. The restoration of angiopoietin-1 (Ang-1) and nuclear factor-E2-related factor 2 (Nrf2) levels was also observed prior to the enhancement of downstream signaling of the tyrosine-protein kinase receptor (Tie-2). Most significantly, treatment of diabetic mice with BV dramatically restored the levels of antioxidant enzymes and chemokines and subsequently rescued the macrophages from the triggered mitochondrial apoptosis [49].

In diabetic rats, the BV-loaded wound dressing consisting of 10% polyvinyl alcohol, 0.6% chitosan, and 4% BV was studied. The results showed that the wound dressing improved wound healing and had an anti-inflammatory effect. At the same time, the wound tissues covered with this preparation displayed higher hydroxyproline and glutathione levels and lower IL- 6 levels compared to the control. Thus, the BV-loaded dressing improved wound healing and anti-inflammatory activity (Table 1) [50]. BV therapy significantly increased wound closure in diabetic animal models by improving collagen production and restoring inflammatory cytokine, TGF-β, and VEGF (Table 1) [51].

In a mouse model, nanofibrous honey, polyvinyl alcohol, and chitosan nanofibrous loaded with bee venom (HPCS-BV) were tested, wherein bactericidal and bacteriostatic effects on *Escherichia coli* were observed at a similar level to the commercial Aquacel Ag (ConvaT, Wales, United Kingdom), a commercial antibacterial agent that was utilized as the positive control. On the other hand, HPCS-BV was found to have antibacterial activities against *Staphylococcus aureus* that were superior to Aquacel Ag. Meanwhile, the HPCS-BV nanofibers showed no antibacterial action against *Pseudomonas aeruginosa*, contrary to the antibacterial activity of the Aquacel Ag nanofibers [52].

### 2.5. Facial Wrinkles

Facial wrinkles are a small crease in the skin, especially the face, which is a natural manifestation of ageing. The skin is the human body’s largest organ and is that which is most shown. Chronic exposure to the sun, particularly to bright beams, and ageing are two predisposition factors that cause wrinkles [53,54]. The reduction in collagen production explains the creation of wrinkles and the reduction of skin flexibility. The need for effective remedies to combat facial wrinkles has contributed to a vast number of products to improve skin appearance. Cosmeceuticals are skincare products that are applicable as cosmetics as well as medications. Many cosmeceuticals have been combined with many naturally derived ingredients, and BV is one of them. BV serum has been reported to cease facial wrinkles by clinically decreasing the total area of the wrinkles, count, and size. In clinical voluntary trials, 22 mature Korean women aged 30 and 49 years were supplied with BV facial serum at a concentration of 0.006%. They used 4 mL of the cream twice daily for 12 weeks. According to this study, the application of BV-containing cosmetics was safe, successful, and with negligible irritation [5]. BV serum was proposed as a cosmeceutical to delay the formation of wrinkles, although little research has been done on the anti-wrinkle effects of cosmetics containing BV [5]. Moreover, the application of bvPLA2-free BV in cosmetic products appears to be promising strategies in preventing skin wrinkling and protecting exposure to UVB (Table 1) [55].

### 2.6. Vitiligo

Vitiligo is a pigment disorder characterized by skin and hair depigmentation. Melanocytes are the specific responsible skin pigmentation cells. The re-pigmentation involves the proliferation of melanocytes and/or migration from adjacent normal epidermis or hair follicles, and it is a difficult and prolonged process. Although exposure to ultraviolet rays (UVR) in patients with vitiligo has been considered a safe and effective method of re-pigmentation, new modalities are needed [56,57].

In vitro study showed that BV induced dose-dependent melanocyte proliferation at concentrations of 10 μg/mL or higher for 7 days, resulting in an approximately twofold increase in the number of melanocytes compared to the control (Table 1) [58]. In an animal study, BV mediated melanogenesis by increased production of tyrosinase and activation of protein kinase A (PKA), extracellular signal-regulated kinases (ERK), and phosphatidylinositol 3-kinase (PI3K)/Akt. BV and its ingredients, bvPLA2 and histamine, mediated the melanocyte migration [58,59]. The activation of the H2 receptor by histamine via complicated ERK, CREB, and Akt signaling has been shown to induce the proliferation and migration of normal melanocytes. In culture, histamine caused vitiliginous keratinocytes to die, but not regular keratinocytes [60]. BV histamine and PLA2 have been shown to be successful in cases of vitiligo [58,59].

### 2.7. Psoriasis

Psoriasis is an inflammatory and proliferative skin disease with a heterogeneous genetic background. Psoriasis is characterized by chronic, sharply demarcated, dull red scaly plaques on the skin, particularly the extender and scalp region [61].

Twenty five patients between the ages of 18 and 60 with recalcitrant localized plaque psoriasis (RLPP) were treated with BV. The venom was administered once weekly for 3 months starting with a dosage of 0.05–0.1 mL, and then gradually increased to 1 mL for each injection. Complete response was achieved in 92% of patients in the BV group. Few side effects were observed and managed, i.e., erythema, moderate pain, and swelling (mild reaction) at the BV injection site. Use of BV therapy is currently available after the advance in BV collecting apparatus [62,63].

A significant number of papers have already been published dealing with BV chemistry, showing the variability of its chemical composition depending on different honeybee strains, geographical changes, and the period and season of venom collection. Therefore, a better understanding on the venom’s biology, chemistry, and its constituents is required to help a safe usage of the BV products [64].

**Table 1:** Bee venom and its bioactive components that are used to treat skin diseases.

Skin Diseases	Model	Dose Used	Mechanism	References
Atopic dermatitis (AD)	Male HR-1 mice	0.1, 0.25, and 0.5 µg of BV	Inhibits the activation of NF-κB and resulting in the reduction in pro- inflammatory cytokines TNF-α, IL-1β, and IL-6. The inhibition of iNOS and COX- 2 expression in a PA-induced AD animal model in a dose-dependent manner.	[21]
	Male BALB/c mice	0.3 mg/kg daily at BL40 acupuncture points for 5 days from BV	Inhibits the proliferation and infiltration of T cells, the production of Th1 and Th2 cytokines, and the synthesis of IL-4 and IgE—typical allergic Th2 responses in blood.	[22]
	Male BALB/c mice	0.01 and 0.1 mg/kg of BV	Inhibits the mast cell degranulation and pro-inflammatory cytokine expression via NF-κB activation.	[24]
	A human keratinocyte cell line, HaCaT cells	0.1 µg/mL of apamin	Inhibits TNF-α- and IFN-γ-induced pro-inflammatory cytokines and Th2 lymphocyte chemokines via down-regulation of NF-κB signaling pathway and STAT.	
Acne Vulgaris	HaCaT and THP-1 cells	1, 10, and 100 ng/mL of BV	Has anti-inflammatory properities against *Propionibacterium acnes*. Blocked TLR2 expression and suppressed the production of IFN-γ, IL-1β, IL-8, and TNF-α induced by *P. acnes*.	[38]
Androgenetic alopecia (AGA)	C57BL/6 mice	0.005% and 0.01% BV	Stimulate the expression levels of growth FGF-2, IGF-1R, and VEG.	[46]
Wound healing	Sprague-Dawley rats	6% *w*/*w* of BV into chitosan film	In comparison to chitosan-free films, the combination had a better anti-inflammatory impact.	[48]
	Adult male Wistar albino	4 % BV incorporated in hydrogel prepared from 10% PVA and 0.6% chitosan	In comparison to the control, had increased hydroxyproline and glutathione levels and lower IL-6 levels.	[50]
	Male BALB/c mice	50 µL equivalent to 200 µg/kg of BV/wounded area/day for 15 days	Significantly restored ATF-3- and iNOS-mediated oxidative stress and MMP-9 expression, as well as enhanced CXCL12-mediated migration EPCs to damaged tissues.	[51]
	Male mice	HPCS-BV nanofibers at different time intervals (3, 5, 7, 10, and 12 days)	Improved collagen deposition and the overall wound-healing process by preventing the inflammatory phase from extending.	[52]
Facial Wrinkles	Twenty-two mature Korean women	BV facial serum at a concentration of 0.006%.	The total wrinkle area, total wrinkle count, and average wrinkle depth were all reduced in clinical trials; however, the exact mechanism is uncertain.	[5]
	Human keratinocyte (HaCaT) and human dermal fibroblast (HDF) cells	1 μg/mL of bvPlA2-free BV	Under UVB exposure, repair cell damage and collagen formation while inhibiting MMP-1 and -13 in HaCaT cells and MMP-1, -2, and -3 in HDF cells. Activation of ERK1/2 and p38 exerts anti-wrinkle properties.	[55]
Vitiligo	Normal human epidermal melanocyte	10 µg/mL BV for 1, 3, 5 or 7 days	Induces cAMP production in melanocytes. Proliferation of melanocytes increased.	[58]
Psoriasis	Twenty-five patients	The doses starting 0.05–0.1 mL/once for a week, and then increased gradually by 0.05 mL every session to a dose of 1 mL for every injection was reached. The total treatment period was 3 months.	Compared to the control group, there was a statistically significant decrease in TNF-α.	[63]

IgE: immunoglobulin E; IL: interleukin; FGF-2: fibroblast growth factor 2; IGF-1R: insulin-like growth factor 1 receptor; VEGF: vascular endothelial growth factor; ATF-3: activating transcription factor-3; iNOS: inducible nitric oxide synthase; MMP-9: matrix metalloproteinase-9; EPCs: endothelial progenitor cells; HPCS: honey, polyvinyl alcohol, chitosan; HaCaT: human keratinocyte; NF-κB: nuclear factor κB; TNF-α: tumor necrosis factor-α; COX-2: cyclooxygenase-2; Th1: T helper cell type 1; Th2: T helper cell type 2; TLR2: toll-like receptor 2; MMP: matrix metalloproteinase; STAT: signal transducer and activator of transcription; CXCL12: C-X-C motif chemokine 12; PVA: poly- vinyl alcohol.

## 3. Concluding Remarks

There is a developing interest in the applications of BV in the cosmetics field, with few formulations commercially available for skin care, atopic dermatitis, and acne. Nevertheless, the cosmetic products available on the market do not point out precisely the BV content and specific components. Efforts should be directed to balance the benefits of using BV with the drawbacks that can induce allergy. Equally interesting, the venom has been used in clinical studies to treat plaque psoriasis, despite the few concerns regarding BV allergic reactions. A critical challenge is the safety of the venom application. Therefore, the venom standardization in addition to the alternative strategies is warranted. VIT is a highly efficient treatment and is strongly recommended to avoid additional sting reactions [65]. VIT is recommended according to the tolerance recognized in beekeepers and populations exposed to bee stings on a regular basis. Beekeepers and their family members are the most exposed to honeybee stings; however, the measurement of specific immunoglobulins in their blood has indicated a higher level of BV-specific IgG and lowered specific IgE compared to the allergic patients. The safety of bee venom immunotherapy is higher in beekeepers than in allergic controls, although the efficacy is comparable in both [66,67]. New strategies to improve the safety and efficacy of the VIT with a reduction of injections would, therefore, be of general interest. Recently, some formulations such as microspheres (biodegradable polymers) containing lyophilized whole venom, aqueous pure venom, and aluminum hydroxide (Al[OH]_3_)-adsorbed preparations have become available in Europe, whereas in the United States, only the formulated whole-venom preparations are approved [68]. Liposomes have a long tradition in drug delivery because they increase the therapeutic index and avoid drug degradation and secondary effects [69].

Refining BV components by purification, modification, or even nanotechnology may potentially limit their toxicity and inhibit harmful side effects. Many successful attempts have been conducted; however, more significant efforts are needed to include BV reliably in drug production [70]. Lastly, in-depth research on BV dermal applications, including cytotoxic and phototoxic effects, as well as preclinical and clinical studies, should be conducted. The involvement of BV in more commercial and conventional primary care products will open new doors of possibilities.

## Data Availability

No new data were created or analyzed in this study. Data sharing is not applicable to this article.

## References

[1] Aufschnaiter A., Kohler V., Khalifa S., El-Wahed A., Du M., El-Seedi H., Büttner S. (2020). Apitoxin and its components against cancer, neurodegeneration and rheumatoid arthritis: Limitations and possibilities. Toxins.

[2] Al-Safar M.A., Obied H.N., Ghaleb R.A., Kashkol A.S. (2020). In-vitro cytotoxic anticancer effects of honeybee venom fractions on different cell lines. Int. J. Drug Deliv. Technol..

[3] Mohamed D., Tedawy E.L., Mahmoud M., Alhaseeb A.B.D., Helmy M.W., Ghoneim A.I. (2020). Systemic bee venom exerts anti-arthritic and anti-inflammatory properties in a rat model of arthritis. Biomed. Rep..

[4] Flávia A., Pereira M., Albano M., Cristina F., Alves B., Fernanda B., Teles M., Furlanetto A., Mores V.L. (2020). Influence of apitoxin and melittin from *Apis mellifera* bee on *Staphylococcus aureus* strains. Microb. Pathog..

[5] Han S.M., Hong I.P., Woo S.O., Chun S.N., Park K.K., Nicholls Y.M., Pak S.C. (2015). The beneficial effects of honeybee-venom serum on facial wrinkles in humans. Clin. Interv. Aging.

[6] Shah S., Gupta A., Karne S.P., Kamble S., Shinde B. (2017). Anti-inflammatory activity of sting protein from *Apis mellifera*. Int. J. Life Sci. Sci. Res..

[7] Han S.M., Kim J.M., Hong I.P., Woo S.O., Kim S.G., Jang H.R., Pak S.C. (2016). Antibacterial activity and antibiotic-enhancing effects of honeybee venom against methicillin-resistant *Staphylococcus aureus*. Molecules.

[8] Han S.M., Lee G.G., Park K.K. (2012). Skin sensitization study of bee venom (*Apis mellifera* L.) in guinea pigs. Toxicol. Res..

[9] Matysiak J., Schmelzer C.E.H., Neubert R.H.H., Kokot Z.J. (2011). Characterization of honeybee venom by MALDI-TOF and nanoESI-QqTOF mass spectrometry. J. Pharm. Biomed. Anal..

[10] Bonifazi F., Jutel M., Biló B.M., Birnbaum J., Muller U. (2005). Prevention and treatment of hymenoptera venom allergy: Guidelines for clinical practice. Allergy.

[11] Müller U.R., Haeberli G. (2005). Use of β-blockers during immunotherapy for Hymenoptera venom allergy. J. Allergy Clin. Immunol..

[12] Diwakar L., Ewan P., Huber P.A.J., Clark A., Nasser S., Krishna M.T. (2016). The impact of national guidelines on venom immunotherapy practice in the United Kingdom. Clin. Exp. Allergy.

[13] Čerpes U., Arzt-Gradwohl L., Schrautzer C., Koch L., Bokanovic D., Laipold K., Tripolt P., Binder B., Sturm G.J. (2020). Simultaneous up-dosing of bee and vespid venom immunotherapy is safe. Allergy Eur. J. Allergy Clin. Immunol..

[14] Tanei R. (2009). Atopic dermatitis in the elderly. Inflamm. Allergy Drug Targets.

[15] Bin L., Leung D.Y.M. (2016). Genetic and epigenetic studies of atopic dermatitis. Allergy Asthma Clin. Immunol..

[16] Giavina-Bianchi M., Giavina-Bianchi P. (2019). Systemic treatment for severe atopic dermatitis. Arch. Immunol. Ther. Exp..

[17] Ferrucci S., Tavecchio S., Berti E., Angileri L. (2021). Dupilumab and prurigo nodularis-like phenotype in atopic dermatitis: Our experience of efficacy. J. Dermatolog. Treat..

[18] Singh R., Heron C.E., Ghamrawi R.I., Strowd L.C., Feldman S.R. (2020). Emerging role of janus kinase inhibitors for the treatment of atopic dermatitis. ImmunoTargets Ther..

[19] Wegner J., Weinmann-menke J., von Stebut E. (2017). Immunoadsorption for treatment of severe atopic dermatitis. Atheroscler. Suppl..

[20] Jin Y., Myung L., Oh J., Hun D., Yong L., Lee S., Lee J., Hyun D., Cheol K., Choi H. (2020). Anti-inflammatory effect of bee venom in phthalic anhydride—Induced atopic dermatitis animal model. Inflammopharmacology.

[21] Oh M.J., Song H.-S. (2020). Anti-Inflammatory effects of bee venom on phthalic anhydride-induced atopic dermatitis. J. Acupunct. Res..

[22] Sur B., Lee B., Yeom M., Hong J.-H., Kwon S., Kim S.-T., Lee H.S., Park H.-J., Lee H., Hahm D.-H. (2016). Bee venom acupuncture alleviates trimellitic anhydride-induced atopic dermatitis-like skin lesions in mice. BMC Complement. Altern. Med..

[23] Kim W., An H., Kim J., Gwon M., Gu H., Sung W.J., Han S.M., Pak S.C., Kim M., Park K. (2017). Beneficial effects of melittin on ovalbumin-induced atopic dermatitis in mouse. Sci. Rep..

[24] Kim K., Lee W., An H., Kim J., Chung H., Han S., Lee K., Pak S.C., Park K. (2013). Bee venom ameliorates compound 48 / 80-induced atopic dermatitis-related symptoms. Int. J. Clin. Exp. Pathol..

[25] Kim W.-H., An H.-J., Kim J.-Y., Gwon M.-G., Gu H., Lee S.-J., Park J.Y., Park K.-D., Han S.-M., Kim M.-K. (2017). Apamin inhibits TNF-α- and IFN-γ-induced inflammatory cytokines and chemokines via suppressions of NF-κB signaling pathway and STAT in human keratinocytes. Pharmacol. Rep..

[26] You C.E., Moon S.H., Lee K.H., Kim K.H., Park C.W., Seo S.J., Cho S.H. (2016). Effects of emollient containing bee venom on atopic dermatitis: A double-blinded, randomized, base-controlled, multicenter study of 136 patients. Ann. Dermatol..

[27] An H.J., Kim J.Y., Kim W.H., Gwon M.G., Gu H.M., Jeon M.J., Han S.M., Pak S.C., Lee C.K., Park I.S. (2018). Therapeutic effects of bee venom and its major component, melittin, on atopic dermatitis in vivo and in vitro. Br. J. Pharmacol..

[28] Jung K.H., Baek H., Kang M., Kim N., Lee S.Y., Bae H. (2017). Bee venom phospholipase A2 ameliorates house dust mite extract induced atopic dermatitis like skin Lesions in mice. Toxins.

[29] Williams H.C., Dellavalle R.P., Garner S. (2012). Acne vulgaris. Lancet.

[30] Zhou M., Xie H., Cheng L., Li J. (2016). Clinical characteristics and epidermal barrier function of papulopustular rosacea: A comparison study with acne vulgaris. Pak. J. Med. Sci..

[31] Jappe U. (2003). Pathological mechanisms of acne with special emphasis on Propionibacterium acnes and related therapy. Acta Derm. Venereol..

[32] Leccia M.T., Auffret N., Poli F., Claudel J.P., Corvec S., Dreno B. (2015). Topical acne treatments in Europe and the issue of antimicrobial resistance. J. Eur. Acad. Dermatol. Venereol..

[33] Nakase K., Nakaminami H., Takenaka Y., Hayashi N., Kawashima M., Noguchi N. (2014). Relationship between the severity of acne vulgaris and antimicrobial resistance of bacteria isolated from acne lesions in a hospital in Japan. J. Med. Microbiol..

[34] Dessinioti C., Katsambas A.D. (2010). The role of Propionibacterium acnes in acne pathogenesis: Facts and controversies. Clin. Dermatol..

[35] Qin M., Pirouz A., Kim M.-H., Krutzik S.R., Garbán H.J., Kim J. (2014). *Propionibacterium acnes* induces IL-1β secretion via the NLRP3 inflammasome in human monocytes. J. Investig. Dermatol..

[36] Jugeau S., Tenaud I., Knol A.C., Jarrousse V., Quereux G., Khammari A., Dreno B. (2005). Induction of toll-like receptors by *Propionibacterium acnes*. Br. J. Dermatol..

[37] Jahns A.C., Lundskog B., Ganceviciene R., Palmer R.H., Golovleva I., Zouboulis C.C., McDowell A., Patrick S., Alexeyev O.A. (2012). An increased incidence of *Propionibacterium acnes* biofilms in acne vulgaris: A case-control study. Br. J. Dermatol..

[38] Kim J.Y., Lee W.R., Kim K.H., An H.J., Chang Y.C., Han S.M., Park Y.Y., Pak S.C., Park K.K. (2015). Effects of bee venom against *Propionibacterium acnes*-induced inflammation in human keratinocytes and monocytes. Int. J. Mol. Med..

[39] An H.J., Lee W.R., Kim K.H., Kim J.Y., Lee S.J., Han S.M., Lee K.G., Lee C.K., Park K.K. (2014). Inhibitory effects of bee venom on *Propionibacterium acnes*-induced inflammatory skin disease in an animal model. Int. J. Mol. Med..

[40] Han S.M., Pak S.C., Nicholls Y.M., Macfarlane N. (2016). Evaluation of anti-acne property of purified bee venom serum in humans. J. Cosmet. Dermatol..

[41] Han S.M., Lee K.G., Pak S.C. (2013). Effects of cosmetics containing purified honeybee (*Apis mellifera* L.) venom on acne vulgaris. J. Integr. Med..

[42] Adil A., Godwin M. (2017). The effectiveness of treatments for androgenetic alopecia: A systematic review and meta-analysis. J. Am. Acad. Dermatol..

[43] Gan D.C.C., Sinclair R.D. (2005). Prevalence of male and female pattern hair loss in Maryborough. J. Investig. Dermatol. Symp. Proc..

[44] Varothai S., Bergfeld W.F. (2014). Androgenetic alopecia: An evidence-based treatment update. Am. J. Clin. Dermatol..

[45] Jain R., De-Eknamkul W. (2014). Potential targets in the discovery of new hair growth promoters for androgenic alopecia. Expert Opin. Ther. Targets.

[46] Park S., Erdogan S., Hwang D., Hwang S., Han E.H., Lim Y.-H. (2016). Bee venom promotes hair growth in association with inhibiting 5α-reductase expression. Biol. Pharm. Bull..

[47] Han S.-M., Lee K.-G., Yeo J.-H., Kim W.-T., Park K.-K. (2011). Biological effects of treatment of an animal skin wound with honeybee (*Apis melifera* L.) venom. J. Plast. Reconstr. Aesthetic Surg..

[48] Amin M.A., Madkor H.R. (2008). Wound healing and anti-inflammatory activities of bee venom-chitosan blend films. J. Drug Deliv. Sci. Technol..

[49] Hozzein W.N., Badr G., Badr B.M., Allam A., Al A., Al-wadaan M.A., Al-waili N.S. (2018). Bee venom improves diabetic wound healing by protecting functional macrophages from apoptosis and enhancing Nrf2, Ang-1 and Tie-2 signaling. Mol. Immunol..

[50] Amin M.A., Abd-Raheem I. (2014). Accelerated wound healing and anti-inflammatory effects of physically cross linked polyvinyl alcohol-chitosan hydrogel containing honey bee venom in diabetic rats. Arch. Pharm. Res..

[51] Badr G., Hozzein W.N., Badr B.M., Al Ghamdi A., Saad Eldien H.M., Garraud O. (2016). Bee venom accelerates wound healing in diabetic mice by suppressing activating transcription factor-3 (ATF-3) and inducible nitric oxide synthase (iNOS)-mediated oxidative stress and recruiting bone marrow-derived endothelial progenitor cells. J. Cell. Physiol..

[52] Sarhan W.A. (2017). Apitherapeutics and phage-loaded nanofibers as wound dressings with enhanced wound healing and antibacterial activity. Nanomedicine.

[53] Kezic S., Novak N., Jakasa I., Jungersted J.M., Simon M., Brandner J.M. (2014). Skin barrier in atopic dermatitis. Front. Biosci..

[54] Hord N.G., Fenton J.I. (2007). Context is everything: Mining the normal and preneoplastic microenvironment for insights into the diet and cancer risk conundrum. Mol. Nutr. Food Res..

[55] Lee H., Kyeong S., Pyo B.M., Heo Y., Goo C. (2015). Anti-wrinkle effect of PLA 2 -free bee venom against UVB-irradiated human skincells. J. Agric. Life Sci..

[56] Bastonini E., Bellei B., Filoni A., Kovacs D., Iacovelli P., Picardo M. (2019). Involvement of non-melanocytic skin cells in vitiligo. Exp. Dermatol..

[57] Roberts G.H.L., Santorico S.A., Spritz R.A. (2019). The genetic architecture of vitiligo. Pigment. Cell Melanoma Res..

[58] Jeon S., Kim N.H., Koo B.S., Lee H.J., Lee A.Y. (2007). Bee venom stimulates human melanocyte proliferation, melanogenesis, dendricity and migration. Exp. Mol. Med..

[59] Maeda K., Tomita Y., Naganuma M., Tagami H. (1996). Phospholipases induce melanogenesis in organ-cultured skin. Photochem. Photobiol..

[60] Kim N.H., Lee A.Y. (2010). Histamine effect on melanocyte proliferation and vitiliginous keratinocyte survival. Exp. Dermatol..

[61] Takeshita J., Grewal S., Langan S.M., Mehta N.N., Ogdie A., Van Voorhees A.S., Gelfand J.M. (2017). Psoriasis and comorbid diseases: Epidemiology. J. Am. Acad. Dermatol..

[62] El-Wahed A.A.A., Khalifa S.A., Sheikh B.Y., Farag M.A., Saeed A., Larik F.A., Koca-Caliskan U., AlAjmi M.F., Hassan M., Wahabi H.A. (2019). et al. Bee venom composition: From chemistry to biological activity. Studies in Natural Products Chemistry.

[63] Eltaher S., Mohammed G.F., Younes S., Elakhras A. (2015). Efficacy of the apitherapy in the treatment of recalcitrant localized plaque psoriasis and evaluation of tumor necrosis factor-alpha (TNF-α) serum level: A double-blind randomized clinical trial. J. Dermatol. Treat..

[64] Kokot Z.J., Matysiak J., Urbaniak B., Dereziński P. (2011). New CZE-DAD method for honeybee venom analysis and standardization of the product. Anal. Bioanal. Chem..

[65] Ridolo E., Pellicelli I., Kihlgren P., Nizi M.C., Pucciarini F., Senna G., Incorvaia C. (2019). Immunotherapy and biologicals for the treatment of allergy to Hymenoptera stings. Expert Opin. Biol. Ther..

[66] Van Vaerenbergh M., Cardoen D., Formesyn E.M., Brunain M., Van Driessche G., Blank S., Spillner E., Verleyen P., Wenseleers T., Schoofs L. (2013). Extending the honey bee venome with the antimicrobial peptide apidaecin and a protein resembling wasp antigen 5. Insect Mol. Biol..

[67] King T.P., Spangfort M.D. (2000). Structure and biology of stinging insect venom allergens. Int. Arch. Allergy Immunol..

[68] Francese S., Turillazzi S., Moneti G., Clench M., Barber D., Kingdom U. (2011). In situ imaging of honeybee (*Apis mellifera*) venom components from aqueous and aluminum hydroxide-adsorbed venom immunotherapy preparations. J. Allergy Clin. Immunol..

[69] Silva T.C., De Paula Moura S., Ramos H.R., De Araujo P.S., Bueno Da Costa M.H. (2008). Design of a modern liposome and bee venom formulation for the traditional VIT-venom immunotherapy. J. Liposome Res..

[70] Ahn Y.J., Shin J.S., Lee J., Lee Y.J., Kim M.R., Shin Y.S., Park K.B., Kim E.J., Kim M.J., Lee J.W. (2016). Safety of essential bee venom pharmacopuncture as assessed in a randomized controlled double-blind trial. J. Ethnopharmacol..

